# Low-frequency stimulation of the primary focus retards positive transfer of secondary focus

**DOI:** 10.1038/s41598-017-00479-z

**Published:** 2017-03-23

**Authors:** Yifang Kuang, Cenglin Xu, Yinxi Zhang, Yi Wang, Xiaohua Wu, Ying Wang, Yao Liu, Kai Zhong, Hui Cheng, Yi Guo, Shuang Wang, Meiping Ding, Zhong Chen

**Affiliations:** 1 Department of Neurology & Epilepsy Center, Second Affiliated Hospital, School of Medicine, Zhejiang University, Hangzhou, China; 2 Department of Pharmacology, Key Laboratory of Medical Neurobiology of the Ministry of Health of China, College of Pharmaceutical Sciences, School of Medicine, Zhejiang University, Hangzhou, China; 3 Department of Neurology, Sir Run Run Shaw Hospital, School of Medicine, Zhejiang University, Hangzhou, China; 4 Collaborative Innovation Center for Diagnosis and Treatment of Infectious Diseases, First Affiliated Hospital, School of Medicine, Zhejiang University, Hangzhou, China

## Abstract

Positive transfer of secondary focus (PTS) refers to new epileptogenesis outside the primary focus and is minimally controlled by existing treatments. Low-frequency stimulation (LFS) has benefits on the onset of epilepsy and epileptogenesis. However, it’s unclear whether LFS can retard the PTS in epilepsy. Here we found that PTS at both contralateral amygdala and ipsilateral hippocampus were promoted after the primary focus was fully kindled in rat kindling model. The promotion of PTS at the mirror focus started when the primary kindling acquisition reached focal seizures. LFS retarded the promotion of PTS when it was applied at the primary focus during its kindling acquisition, while it only slightly retarded the promotion of PTS when applied after generalized seizures. Meanwhile, we found the expression of potassium chloride cotransporter 2 (KCC2) decreased during PTS, and LFS reversed this. Further, the decreased expression of KCC2 was verified in patients with PTS. These findings suggest that LFS may be a potential therapeutic approach for PTS in epilepsy.

## Introduction

The term positive transfer of secondary focus (PTS) refers to epileptogenesis in a naive area induced by repetitively uncontrolled epileptic seizures from a primary seizure focus^1, 2^. Up to 34% of the epilepsy patients experience PTS, the ratio in temporal lobe epilepsy (TLE) is even higher^3^. Existing drug treatments, such as valproate, carbamazepine and lamotrigine usually are ineffective for PTS^4, 5^. Moreover, patients with secondary focus usually have limited outcomes after surgery resection of primary focus^5–8^. Poor control of PTS and its recurrent seizures would put those patients in danger^9–12^. Thus, it is emergent to search a new treatment for PTS.

Low frequency stimulation (≤5 Hz, LFS) is a promising therapeutic strategy for epilepsy. It has the advantages of reversibility, controllability, and minimal invasiveness^13, 14^. Evidence from both clinical and preclinical data has indicated that, when targeting crucial regions, LFS can suppress the severity of seizures^15–19^. Experimental results also support the idea that LFS directly targeting the seizure focus can confer an anti-epileptogenic effect on various epilepsy models, both *in vitro* and *in vivo* ^20–22^. These studies have established that LFS treatment has benefits not only on epileptic seizures onsets, but also on epileptogenesis. Thus, it is possible that LFS may inhibit the formation of secondary epileptogenesis, i.e. PTS. However, PTS can emerge at multiple locations and are often difficult to foresee^5^. This situation makes the potential target sites for LFS extremely difficult to choose.

Recently, our group demonstrated that LFS applied outside the seizure focus, such as at the piriform cortex, cerebellar fastigial nucleus or subiculum, retard epileptogenesis in a kindling model^15, 18, 19^. These lead us to hypothesize that LFS applied at the primary focus (outside the secondary focus, and have neural projection to the secondary focus) may suppress PTS. Thus, in the present study, we aimed to test whether LFS at the primary focus during its kindling acquisition could suppress subsequent PTS in a rat kindling model. Additionally, as previous studies suggested that potassium chloride cotransporter 2 (KCC2) was closely associated with PTS^23^, we further assess whether the anti-epileptic effect of LFS was associated with KCC2.

## Results

### LFS at the primary focus retards the PTS

**Figure 1:**
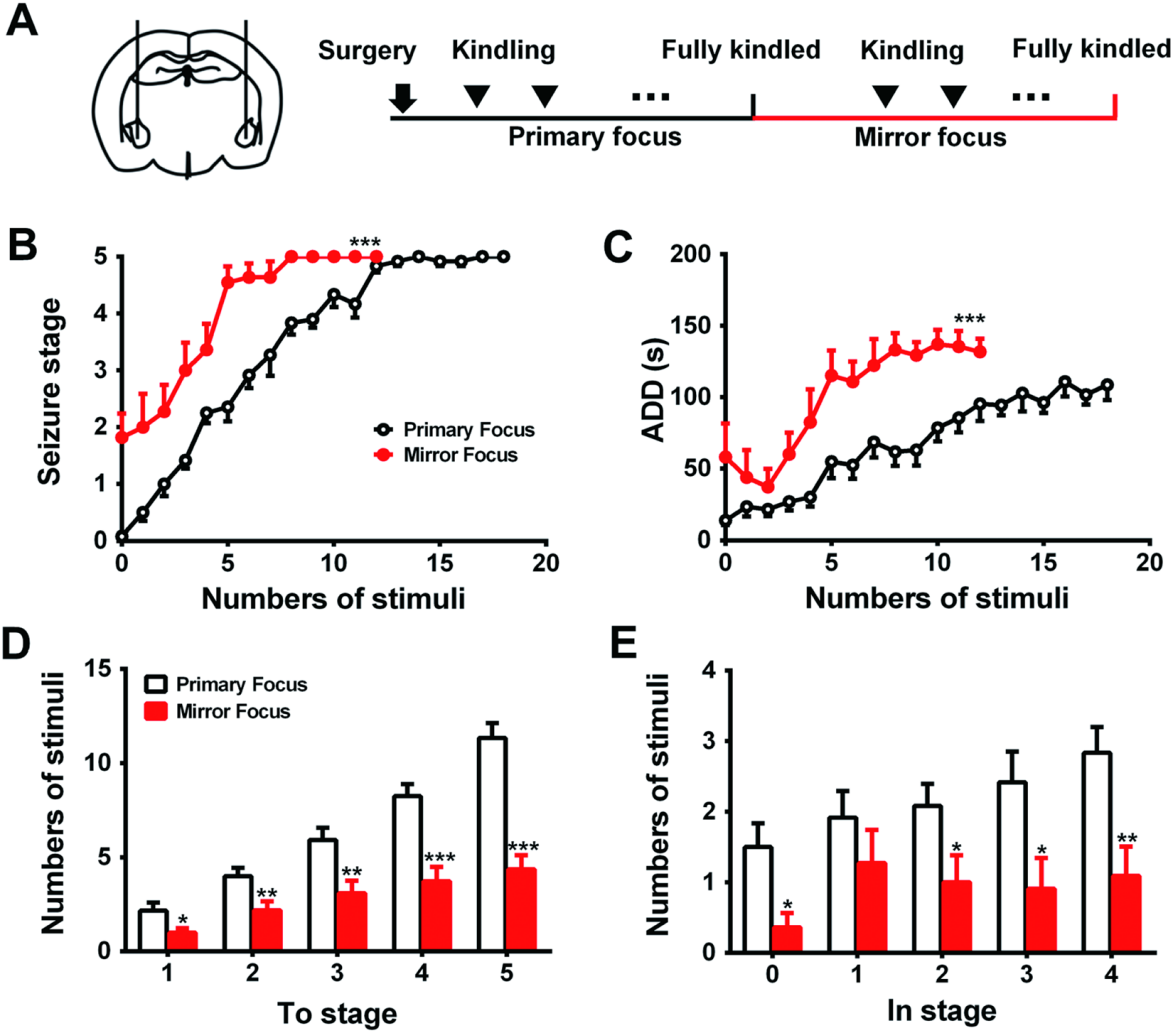
Primary kindling acquisition promoted PTS at the mirror focus. (**A**) Schematic of experiment. (**B**,**C**) The progression of behavioral stages (**B**), and mean ADDs (**C**) during kindling acquisition at the mirror focus, after the primary focus was fully kindled (n = 11 for both groups); (**D**,**E**) numbers of stimulation required to reach each stage (**D**), and numbers of stimulation required to stay in each stage (**E**) during kindling acquisition at the mirror focus. *p < 0.05, **p < 0.01, and ***p < 0.001 represents differences compared with the primary-focus group. Two-way ANOVA with repeated measures was used for statistical analysis of (**B** and **C**). Student’s t test was used for statistical analysis of (**D** and **E**).

After the primary focus (right amygdala, RAM) was fully kindled, we tried to kindle the mirror focus (left amygdala, LAM) (Fig. 1A). We found that the progression of behavioral seizure stage and the mean after-discharge durations (ADDs) in the kindling acquisition of the mirror focus were significantly accelerated and prolonged compared with those of the primary focus (*p* < 0.001 for both, Fig. 1B and C). The number of stimulations to reach each stage of mirror-focus group were significantly less than primary focus group (to stage 1, *p* < 0.05, to stage 2 and 3, *p* < 0.01, to stage 4 and 5, *p* < 0.001, Fig. 1D); the number of stimulations in stage 0, 2, 3 and 4 of mirror-focus group were significantly less than primary focus group (in stage 0, 2 and 3, *p* < 0.05, in stage 4, *p* < 0.01, Fig. 1E). These results demonstrated that PTS at mirror focus was promoted after the primary focus was fully kindled. However, the fully-kindled primary focus is not necessary for the promotion of PTS: the electrical lesion of the kindled primary focus did not alter the progression of seizure stage and ADDs in the mirror focus (*p* > 0.05, Supplementary Fig. 1B and C).

**Figure 2:**
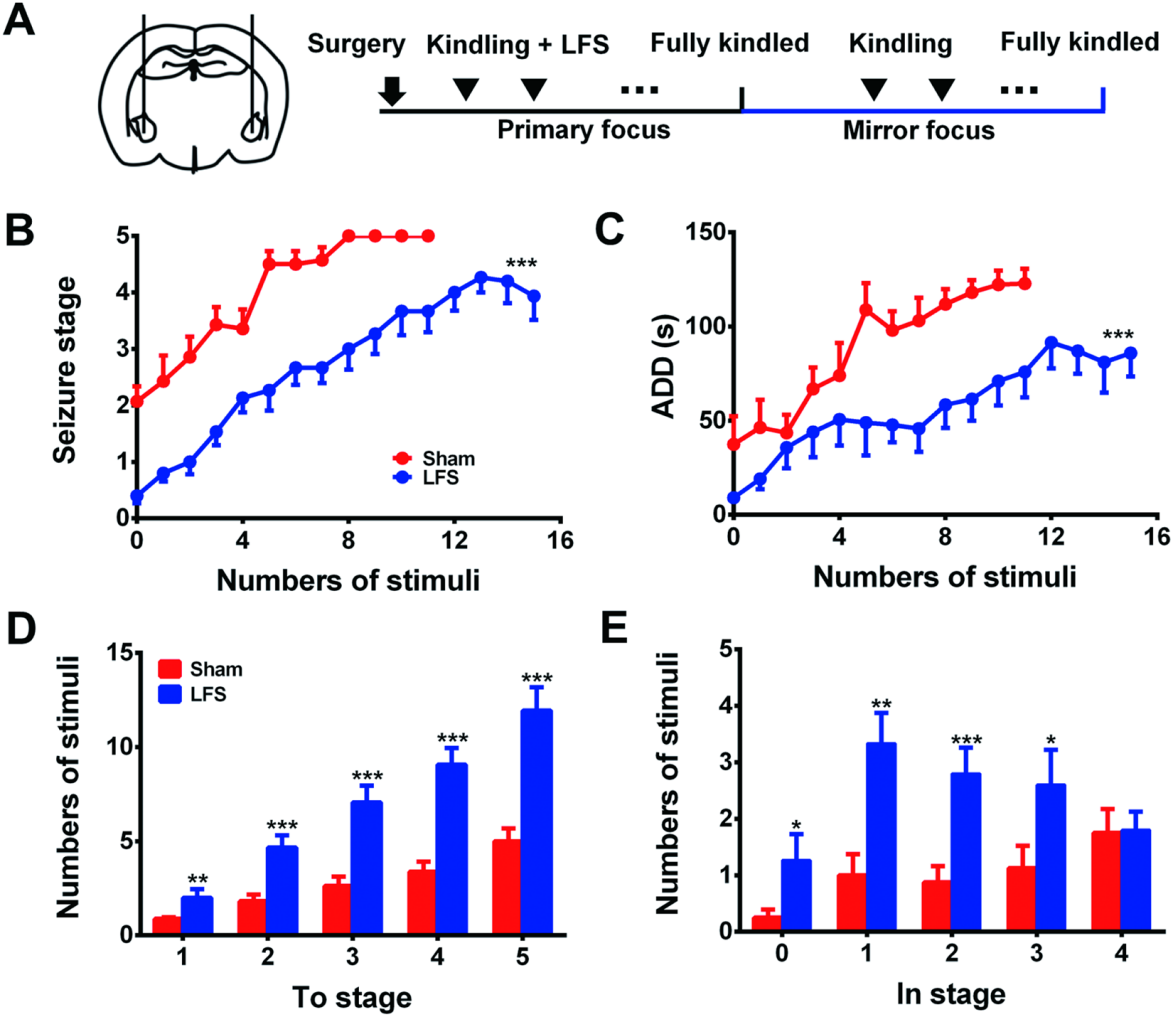
LFS retarded PTS at the mirror focus. (**A**) Schematic of experiments to illustrate the application of LFS. (**B–E**) Effect of LFS targeting the primary focus on seizure stage (**B**), ADD (**C**), numbers of stimulation required to reach each stage (**D**), and numbers of stimulation in each stage (**E**) during kindling acquisition of the mirror focus (n = 16 for Sham group, n = 15 for LFS group). *p < 0.05, **p < 0.01, and ***p < 0.001 represents differences compared with Sham group. Two-way ANOVA with repeated measures was used for statistical analysis of (**B** and **C**). Student’s t test was used for statistical analysis of (**D** and **E**).

To investigate the effect of LFS on PTS, we applied LFS at the primary focus during its kindling acquisition (Fig. 2A). LFS at primary focus could retard the kindling acquisition at the primary focus (p < 0.05, Supplementary Fig. 2). Interestingly, LFS at primary focus also significantly retarded the progression of behavioral seizure stage (*p* < 0.001, Fig. 2B) and shortened the mean ADDs (*p* < 0.001, Fig. 2C) in the kindling acquisition of the mirror focus. It also increased the number of stimulation to reach each stage (to stage 1, *p* < 0.01, to stage 2–5, *p* < 0.001, Fig. 2D), and increased the number of stimulations in stage 0–3 (in stage 0 and 3, *p* < 0.05, in stage 1, *p* < 0.01, in stage 2, *p* < 0.001, Fig. 2E). Furthermore, we tested the PTS at the hippocampus, and found that after the primary focus (amygdala) was fully kindled, the kindling acquisition of ipsilateral hippocampus (red circle line in Supplementary Fig. 3B) was significantly accelerated, compared with the kindling acquisition of hippocampus as the primary focus (black circle line in Supplementary Fig. 3B). These results indicated that LFS at the primary focus could retard the promotion of PTS.

### LFS applied after focal seizure only slightly retards PTS

**Figure 3:**
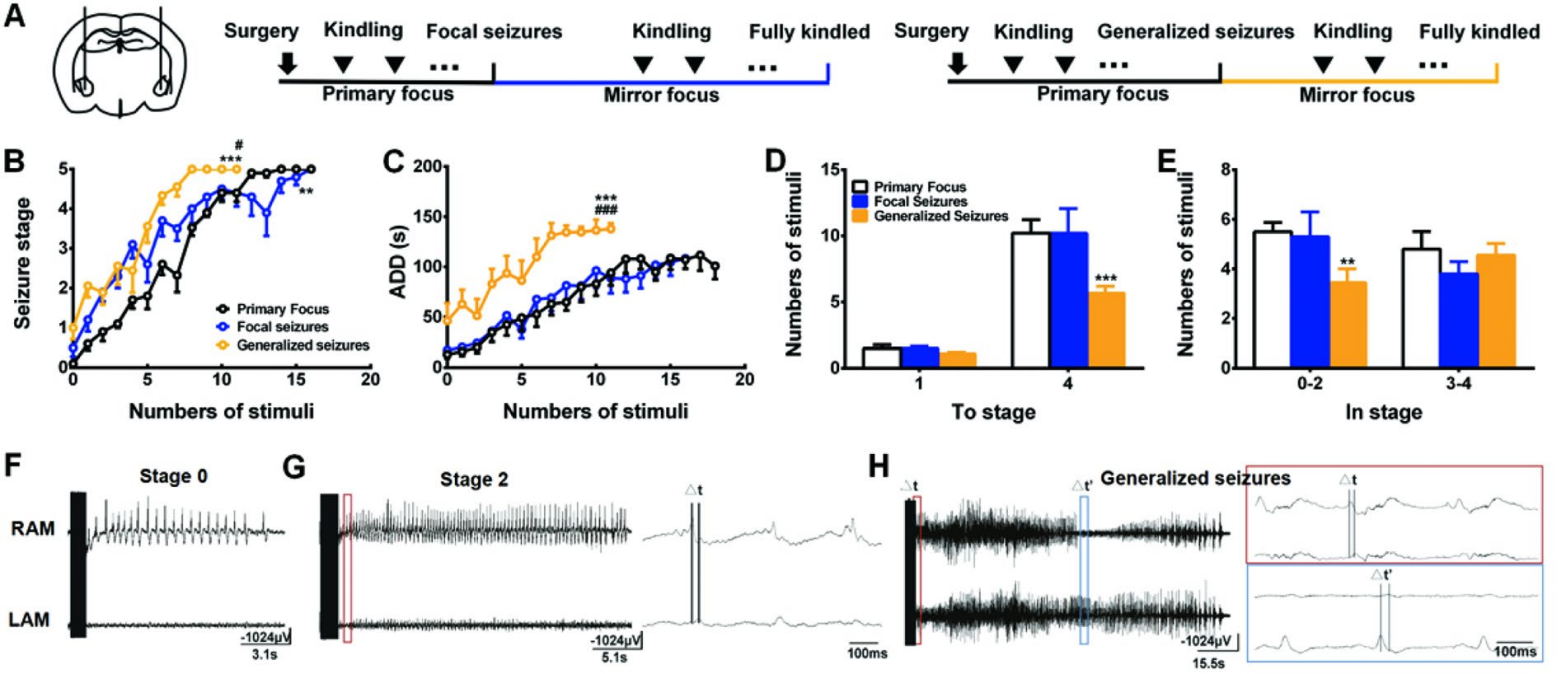
PTS was promoted as early as focal seizure stage of primary kindling acquisition (**A**) Schematic of experiments showing when the progression of PTS in the mirror focus were tested. (**B–E**) The development of seizure stage (**B**), ADDs (**C**), numbers of stimulation required to reach each stage (**D**), and numbers of stimulation in each stage (**E**) during kindling acquisition of the mirror focus, when primary focus reached the focus seizures or generalized seizures (n = 10 for the Primary-Focus group and the Generalized-seizures group, n = 9 for the Focal-seizures group). *p < 0.05, **p < 0.01, ***p < 0.001: compared with the Primary Focus group. ^#^ *p* < 0.05, ^##^ *p* < 0.01, ^###^ *p* < 0.001: compared with the Focal seizures group. Two-way ANOVA followed by LSD *post hoc* tests were used for statistical analysis of (**B** and **C**); One-way ANOVA followed by LSD *post hoc* tests were used for statistical analysis of (**D** and **E**). (**F–H**) Representative EEGs acquired from RAM and LAM during different stages of primary epileptogenesis.

PTS requires recurrent stimulation of the primary focus to activate the naive region other where^23^. To understand when the promotion of PTS started, the progression of PTS in the mirror focus was tested at the time when the primary kindling acquisition reached focal seizures or generalized seizures (Fig. 3A). When the primary kindling acquisition reached focal seizures, the progression of seizure stage in the mirror focus was accelerated compared with the primary kindling acquisition (*p* < 0.01, Fig. 3B), while the mean ADDs had no difference (*p* > 0.05, Fig. 3C). When the primary kindling acquisition reached generalized seizures, the progression of seizure stage (*p* < 0.001, Fig. 3B) and the mean ADDs (*p* < 0.001, Fig. 3C) in the mirror focus were accelerated compared with the primary kindling acquisition. EEG recordings showed that there were no seizures-like spikes in the mirror focus when primary focus received the first kindling stimulation (Fig. 3F). However, when the primary kindling acquisition reached stage 2, these spikes were propagated from the primary focus to the mirror focus (Fig. 3G). Moreover, when the primary kindling acquisition reached generalized seizures, seizure-like spikes originated from mirror focus could be recorded as well (Fig. 3H). These results indicated that PTS at a mirror focus may be promoted as early as focal seizure stage of primary kindling acquisition.

**Figure 4:**
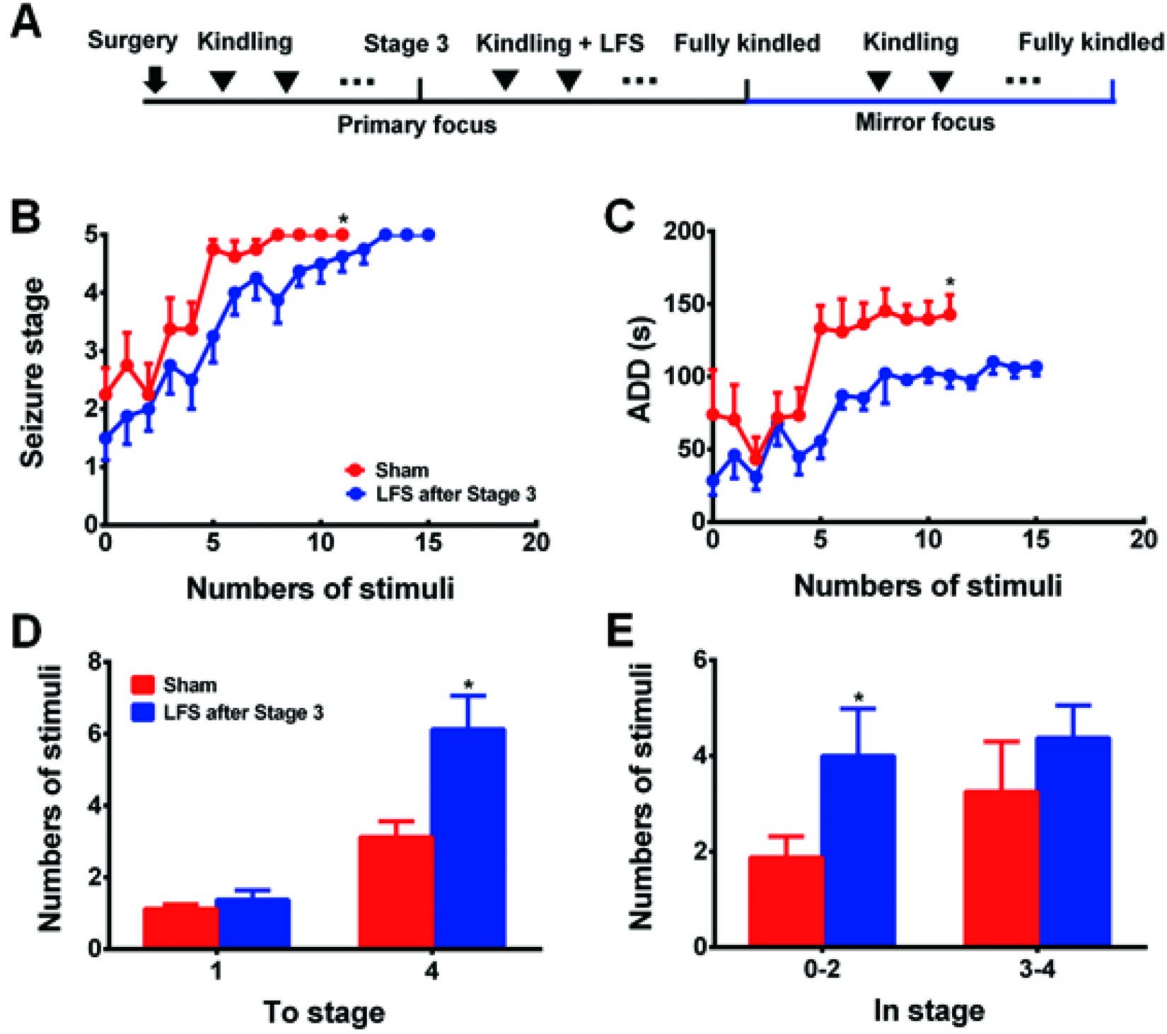
LFS treatment applied after focal seizure stages of primary kindling acquisition slightly retards PTS (**A**) Shematic of experiments showing delivering LFS after the primary kindling reaches stage 3. (**B–E**) Effect of LFS delivering after the primary kindling reaches stage 3 on seizure stage (**B**), ADD (**C**), numbers of stimulation required to reach each stage (**D**), and numbers of stimulation in each stage (**E**) during kindling acquisition of the mirror focus (n = 8 for both groups). *p < 0.05: compared with the Mirror-focus group. Two-way ANOVA with repeated measures was used for statistical analysis of (**B** and **C**). Student’s t tests were used for statistical analysis of (**D** and **E**).

To further test whether there is a time-window for LFS to inhibit PTS, we applied LFS at the primary focus when the primary kindling acquisition reached seizure stage 3 (Fig. 4A). We found that LFS retarded the progression of seizure stage (*p* < 0.05, Fig. 4B) and shortened mean ADDs in the mirror focus compared with Sham group (*p* < 0.05, Fig. 4C). The number of stimulations to stage 4 in LFS group was more than Sham group (*p* < 0.05, Fig. 4D). The number of stimulations in stage 0–2 in LFS group was more than Sham group (*p* < 0.05, Fig. 4E). These results indicated that LFS applied after focal seizure stages in the primary kindling acquisition modestly retarded the promotion of PTS at the mirror focus.

### LFS reversed the decrease in the expression of KCC2 during PTS

**Figure 5:**
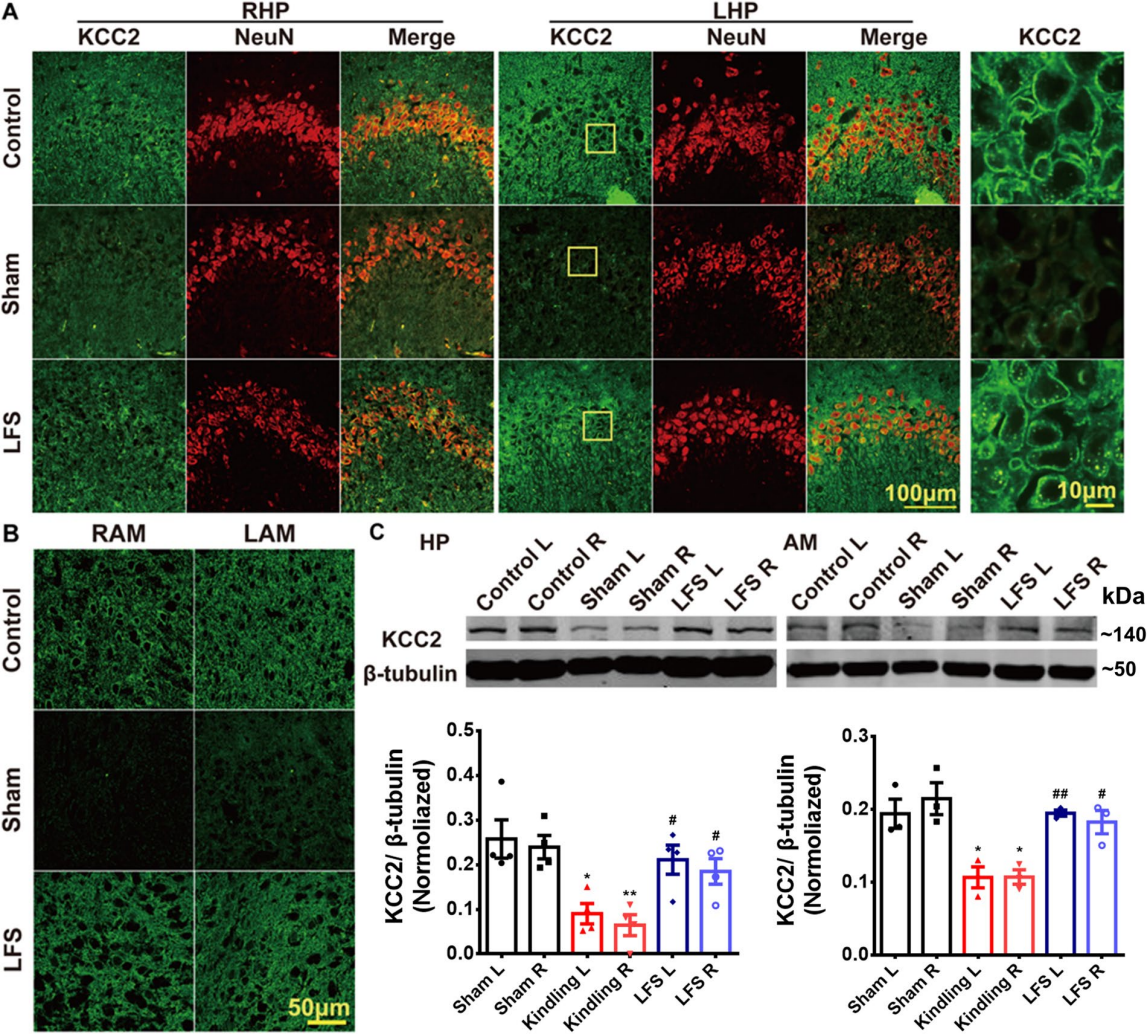
LFS blocked the decreased expression of KCC2 that accompanied with PTS. (**A** and **B**) Coronal sections of bilateral hippocampus (**HP, A**) and amygdala (**AM, B**) from the Control group, Sham group, and LFS group immunolabeled for KCC2 (green) and NeuN (Red). (**C**) Western-blot and densitometric analysis of the expression of the KCC2 protein in the hippocampus and the amygdala when the primary site was fully kindled. Full length blots are shown in Supplementary Figure 6. *p < 0.05, **p < 0.01: compared with the Sham group. ^#^ *p* < 0.05, ^##^ *p* < 0.01: compared with the Kindling group. One-way ANOVA followed by LSD *post hoc* tests were used for statistical analysis of (**C**).

**Figure 6:**
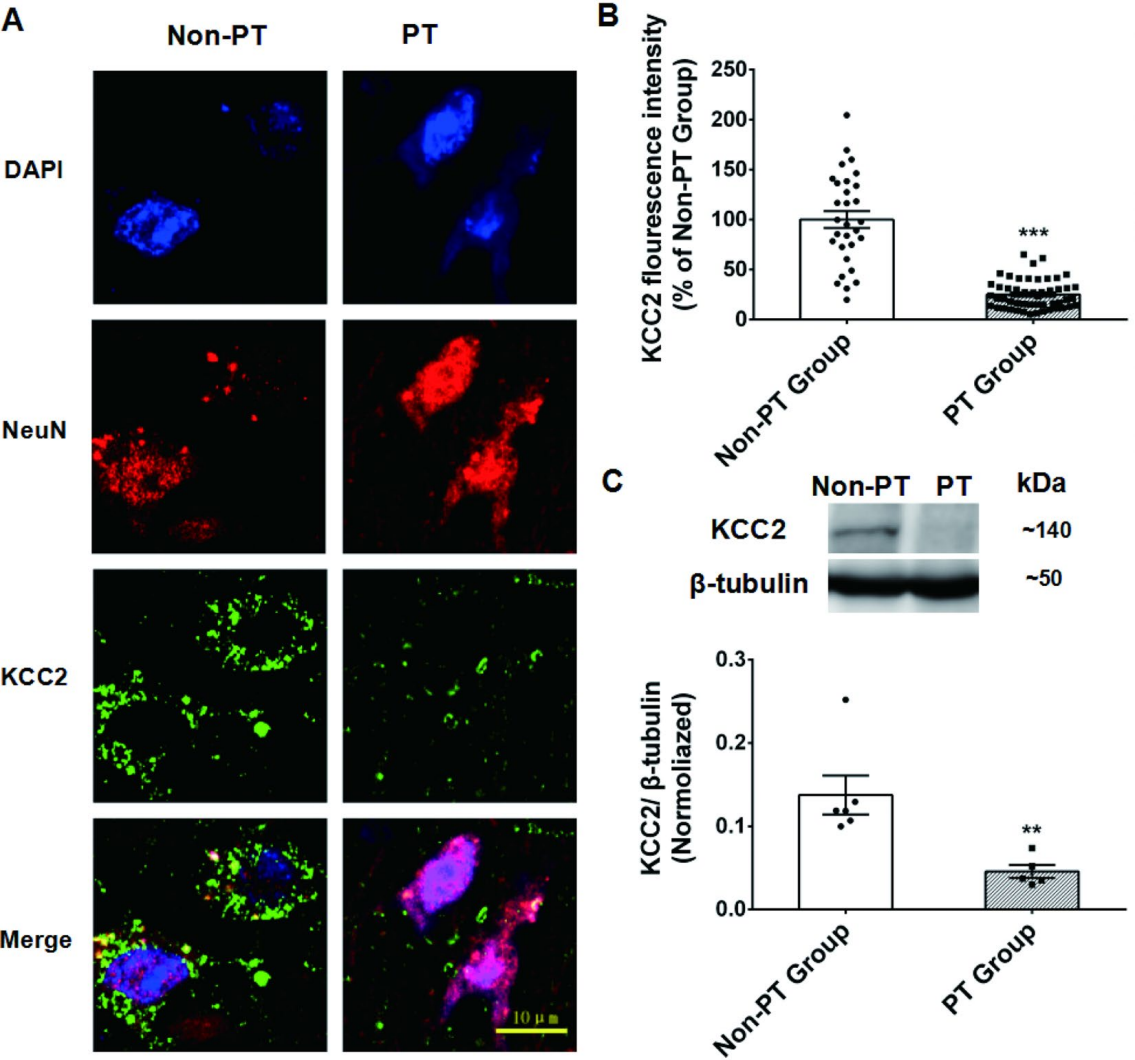
PTS patients showed decreased expression of KCC2. (**A**) KCC2 immunohistochemistry in PT patients (P1) and non-PT (N4) patients. Both sites are resected hippocampus. (**B**) immuno-fluorescent signal intensity per neuron in each patient (10 neurons were analyzed in each patient) were analyzed. (**C**) Western-blot of KCC2 and densitometric analysis in PT patients and non-PT patients, full length blot is shown in Supplementary Figure 7. ***p < 0.001, **p < 0.01 compared with the Non-PT group. Student’s *t* tests were used for statistical analysis of (**B** and **C**).

Previous preclinical study demonstrated that the expression of KCC2 is closely related to PTS at mirror focus^24^. In the present study, we found that in the fully kindled group, the immunoreactivity of KCC2 in both the primary focus and the secondary foci (contralateral amygdala and ipsilateral hippocampus) were lower than control group (Fig. 5A and B). KCC2 was located regularly near the membrane of cell bodies from control group, but point-like distributed in sham group. Western-blotting also confirmed this phenomenon (Fig. 5C), and further showed that the expression of KCC2 only significantly decreased when the kindling acquisition of primary focus reached stage 3–5 (Supplementary Fig. 4). To further verify the decrease of the expression of KCC2 in clinical TLE patient with PTS (Supplementary Tables 1 and 2), we divided patients into positive transfer (PT) group and no positive transfer (Non-PT) group. In our study, 6 PT patients and 7 Non-PT patients were included. The average age, course of epilepsy and seizure frequency showed no significant differences between these two groups (Supplementary Table 3). And we analyzed the KCC2 expression in the surgical removed sample from TLE patients with or without PTS. The results of immunoreactivity (Fig. 6A and B, *p* < 0.001) and western-blotting (*p* < 0.01, Fig. 6C) both showed that in patients with PTS, the expression of KCC2 in the primary focus was lower than that in patients who had only one seizure focus. And sclerosis patients in PT and Non-PT groups both had grade 1 sclerosis (Supplementary Table 4).

In addition, the immunoreactivity of KCC2 in LFS group showed no significant difference compared with control group (Fig. 5A and B). Western-blotting also confirmed this phenomenon (Fig. 5C). These indicated that LFS could reverse the decrease of KCC2 during PTS in rats. However, LFS did not alter the expression of KCC2 in normal rats (Supplementary Fig. 5).

## Discussion

PTS is minimally controlled by existing treatments. In the present study, we found that PTS at contralateral amygdala (mirror focus) was promoted after the primary focus was fully kindled in rats. The fully-kindled primary focus is not necessary for the maintenance of PTS, since PTS is still promoted when the primary focus was lesioned. Also, LFS at the primary focus retarded the promotion of PTS at mirror focus. At present, it has established that LFS treatment has benefits on primary epileptic seizures onsets or primary epileptogenesis. Therefore, our studies broaden the clinical application of LFS by showing that it can inhibit PTS at multiple foci, which made it a promising treatment in drug-resistant PTS patients.

Repetitive uncontrolled epileptic seizures from a primary focus would trigger PTS in other secondary foci^23^. For this reason, defining when PTS would happen becomes very important for the timely application of LFS. It has been reported that there are three phases of PTS: the dependent phase, the intermediate phase and the independent phase^6, 25^. Irreversible transfer in a naive region commonly occurs in the later independent phase^6, 23^. Interestingly, we found that the facilitation of PTS happens during the very early stages of primary epileptogenesis. The accelerated progression of seizure stage at secondary focus began in the focal seizure stage of the primary kindling acquisition, while in this circumstance, ADDs showed no difference. The accelerated progression of ADDs only started from the generalized seizure stage of the primary kindling acquisition. The discrepancy between our findings with others may be due to that previous studies only monitored EEG activity during PTS. Interestingly, when LFS was applied at later stages, we observed limited anti-epileptic effect than that applied during the whole kindling acquisition, indicating that timely LFS treatment is necessary for inhibiting PTS and that the early stage of the primary kindling acquisition might be a critical period for LFS treatment. It provided further evidence to support an anti-epileptic effect on LFS within a critical time-window^26, 27^. The early critical period possibly represents a specific milieu of epileptogenesis, which renders the brain more susceptible to the formation of aberrant neural circuitry that serves to facilitate and strengthen PTS. Blocking the early pathological changes during the critical period of epileptogenesis, may benefit for preventing epilepsy. Thus, according to different phases of PTS, it is important to apply the LFS timely to make a better anti-epileptic effect. To date, no single anti-epileptic drug (AED) can prevent PTS. One possible explanation might be that the AEDs were not administered within the critical period^24^. Thus, we speculated that the early stage might be the crucial period for preventing PTS for both drugs and LFS.

PTS extends to multiple regions outside the primary focus^3^. In the present study, PTS happened at contralateral BLA and ipsilateral hippocampus; both had certain connections with the primary focus (amygdala) by the corpus callosum and hippocampal commissures, respectively. It may be easier to elicit PTS through connected tracts, which is repetitive and persistent neural activity re-organized network^28^. And we found that LFS retarded the promotion of PTS at both secondary foci, indicating that LFS at a primary focus may retard PTS at multiple outside foci. Interestingly, the anti-epileptic effect of LFS on PTS at ipsilateral hippocampus seems to be weaker than that at the amygdala, indicating that different brain structures would influence the effect of LFS. The time required for a secondary focus to develop may be related to cortical complexity. Structures such as the amygdala may be more susceptible to kindling than other structures such as the hippocampus^29^. In addition, in the present study, LFS was applied at the primary focus instead of a secondary focus, because (1) the locations and numbers of PTS events are always unpredictable in a clinical setting^4, 5, 30^, (2) LFS targeted outside of the seizure focus showed promising anti-epileptogenic effects^16, 17^, (3) LFS targeting the primary focus may provide wider coverage of anti-epileptic effects and less additionally invasive lesions. Taken together, these conjectures indicated that LFS targeting the primary focus is a promising approach to inhibit PTS at multiple secondary foci.

GABA-mediated signaling is closely related with epileptic activity and epileptogenesis^28^. Dysfunction of GABAergic neurons increased the frequency of the occurrence of PTS^23^. We examined the levels of a crucial cotransporter, KCC2, which is known to regulate GABAergic neurons^31^ and found that the level of KCC2 is downregulated at both primary and secondary foci. Notably, the expression of KCC2 in patients who experienced PTS was lower than that in patients who had only one seizure focus, indicating that the decrease of KCC2 may be involved in PTS and may be a potential biomarker for PTS. Interestingly, LFS reversed the decreased expression of KCC2 that accompanied with PTS, but did not change the expression of KCC2 in normal rats that received LFS. These observations suggest that LFS modulates the expression of KCC2 in a manner that is dependent on epileptic activities. One proposed mechanism suggests that direct current stimulation (such as kindling stimulation) could accelerate the process that pro-form of BDNF (proBDNF) convert into mature BDNF (mBDNF)^32^. And this conversion of mBDNF needs co-release of proBDNF and tissue plasminogen^33^. In our study, we found that LFS could delay the kindling acquisition process of primary focus, and it might decrease the increased levels of BDNF caused by kindling stimulation at primary focus. And then LFS produce an anti-epileptic effect on PTS. Therefore, we speculated that the anti-epileptic effect of LFS on PTS may be due to modulating the expression of KCC2; a change in KCC2 levels may be a promising biomarker for clinical prediction of PTS in epilepsy. At present, PTS is unpredictable. It may be possible to use KCC2 labeled with radioactive isotopes to provide a new predictive biomarker for PTS.

In conclusion, the present study demonstrated that LFS of the primary focus retards PTS in a rat kindling model. LFS treatment may provide a potential clinical therapeutic approach for PTS in epilepsy.

## Materials and Methods

### Animals

Experiments were performed on male Sprague-Dawley rats (260–330 g, GradeII, Certificate No. SCXK2008–0033, Experimental Animal Center, Zhejiang Academy of Medical Science, Hangzhou, China). All rats were housed under a 12 h light/dark cycle with *ad libitum* feeding and watering. Behavioral experiments were carried out between 8:00 and 17:00. All experiments were approved by the Zhejiang University Animal Experimentation Committee and were in complete compliance with the National Institutes of Health Guide for the Care and Use of Laboratory Animals. The animal data does not address sex as biological variable.

### Patients and preoperative epileptic focus localization

All patients with TLE in this study had typical clinical manifestation and characteristic EEG for epilepsy. None of the patients had a good response to the maximal doses of three or more first-line AEDs, and were regarded as refractory TLE. Thirteen patients (5 males and 8 females) who had undergone epileptic surgery for intractable TLE were recorded in the epilepsy center of the Second Affiliated Hospital of Zhejiang University (Supplementary Tables 1 and 2). And the degree of hippocampus sclerosis was shown in Supplementary Table 4. Informed written consent forms for the use of the tissue in research were obtained prior to surgery. Written informed consent was obtained from both patients with and without PTS and signed by subjects and legal guardians. The research was approved by the Medical Ethical Committee of Zhejiang University School of Medicine and the methods were carried out in accordance with the approved guideline (study number 2012-036). Presurgical assessment included obtaining a detailed history and neurological examination, interictal and ictal scalp EEG analysis (we used 128-channel long-term digital video EEG monitoring and EEG sleep monitoring), neuropsychological testing, and neuroradiological studies, such as brain X-ray computerized tomography (CT) scanning or magnetic resonance imaging (MRI), all these aimed to localize the epileptic foci for each patient. Subdural electrocorticography (ECoG) and invasive deep brain EEG were used to accurately locate the epileptic source and functional areas if necessary. For each patient, on the basis of the evaluation of the detailed electrophysiology and neuroimages, one primary epileptogenic zone (EZ) for resection was identified. Further pathology examination were undertaken to confirm the pre-surgical inspection and pathological type. Patients were grouped depending on whether they had independent secondary epileptiform discharges before surgery. EZ localized in two or more different functional zones were regarded as the PT group, and those with a single EZ were regarded as the Non-PT group. All TLE patients in our study experienced at least one seizure attack within 1 week prior to surgery.

### Amygdala kindling model

According to our previous studies^15, 34–36^, rats were mounted in a stereotaxic apparatus (512600, Stoelting, USA) after pentobarbital sodium anesthesia (45 mg/kg, i.p.), and the electrodes were implanted into the right basolateral amygdala (AP: −2.4 mm, L: −4.8 mm, V: −8.8 mm), the left basolateral amygdala (AP: −2.4 mm, L: 4.8 mm, V: −8.8 mm), or the right hippocampus (AP: −5.3 mm, L: −5.0 mm, V: −6.0 mm) for kindling stimulation and EEG recording. The coordinates were measured from the bregma according to the atlas of *Paxinox and Watson* ^37^. The electrodes were made of twisted stainless-steel wires (diameter 0.125mm, A.M. Systems, USA) insulated except at the tip (0.5 mm) and the tip separation was about 0.5 mm. Two screws were placed in the skull over the cerebellum (AP: −10.5 mm; L: −1.5 mm) to serve as the reference and ground electrodes; the other two screws were placed in the skull before the bregma. All the electrodes and screws were connected to a miniature receptacle, which was attached to the skull with dental cement. Electrode location was histologically verified in all animals following the behavioral studies.

Following 7–10 days of recovery, the after-discharge threshold (ADT) of the right basolateral amygdala (defined as the primary site) in each rat was measured (monophasic square-wave pulses, 60 Hz, 1 ms/pulse, 60 pulses) with a constant current stimulator (SEN-7203, SS-202J; Nihon Kohden, Japan), and EEGs were recorded with a Neuroscan system (Compumedics, Australia). The stimulation intensity started at 60 μA and then increased in 20 μA steps every 30 min. The minimal intensity that produced at least 5-s after-discharge was designated as the ADT for that animal and was used for dividing into different groups and daily stimulation thereafter. The ADT intensity of amygdala ranged from 100 to 400 μA. Seizure severity was classified according to the Racine scale^38^: (1) facial movement; (2) head nodding; (3) unilateral forelimb clonus; (4) bilateral forelimb clonus and rearing; and (5) rearing and falling. Stages 1 and 2 were considered as focal seizures^39^ and stages 3–5 were considered as generalized seizures^40^. Seizure stage was judged by someone who did not know the grouping. When animals had three consecutive stage 5 seizures, they were regarded as fully kindled.

The intracranial EEGs were performed in freely moving rats with band-pass filters spanning DC-200 Hz and sampled at 1000 Hz with a Neuroscan system (Compumedics, Australia).

### LFS treatment at primary focus for PTS

In our animals study, we defined the right amygdala as the primary focus. First, we tested whether the kindling acquisition of primary focus would promote PTS at the contralateral left amygdala (mirror focus, a special form of PTS; this term indicates that the secondary epileptogenic zone can be observed in a contralateral homotopic area to the primary seizure focus). After the primary focus was fully kindled, the ADT of the mirror focus was determined again, and was used for daily kindling stimulation in the mirror focus subsequently (Fig. 1A). We recorded daily seizure stages and ADD, and compared the kindling acquisition of primary focus (primary-focus group) with the mirror focus (mirror-focus group).

To investigate whether PTS is dependent on primary focus, we initiated electrical lesion (constant current, 1 mA, 10 s) of the primary focus after it was fully kindled (Supplementary Fig. 1A). And rats in lesion group and non-lesion group were kindled in the mirror focus, daily seizure stages and ADDs were recorded, and the kindling acquisition of lesion group and non-lesion group were compared.

To test whether LFS at the primary focus can inhibit PTS at the mirror focus, we delivered LFS (monophasic square-wave pulses, 1 Hz, 100 μA, 0.1 ms/pulse for 15 min) immediately after the kindling stimulus at the primary focus via the same electrodes used for kindling. Rats were divided into two groups (LFS group and sham group, Fig. 2A) according to their ADTs. The LFS group received daily LFS until the primary focus was fully kindled, while the sham group received sham LFS (no current delivered). The re-determined ADT of the mirror focus was used thereafter for daily kindling stimulation in the mirror focus experiments for both groups. We recorded the daily seizure stages and ADDs, and compared the kindling acquisition of LFS group with sham group.

### LFS treatment at different seizure stages of primary epileptogenesis for PTS

To determinate the phase when PTS was promoted, the secondary focus began to receive kindling stimulation at different seizure stages during the kindling acquisition of the primary focus (Fig. 3A). Rats were divided into three groups according to their ADTs: focal-seizures group received kindling stimulus at the mirror focus when the right amygdala reached focal seizure stages (stage 1–3); generalized-seizures group received kindling stimulus at mirror focus when the right amygdala reached generalized seizure stages (stage 4–5), but not fully kindled; and primary-focus group received kindling stimulation at left amygdala with no kindling stimulation at the right amygdala.

We further tried to test whether LFS delivered at later stages of kindling acquisition at the primary focus would inhibit PTS at the mirror focus (Fig. 4A). Rats were divided into two groups according to their ADTs: LFS-S3 group received LFS since seizure stage 3 in the kindling acquisition of the primary focus until the primary focus was fully kindled; sham group received sham LFS. The re-determined ADT of the mirror focus was used thereafter for daily kindling stimulation in the mirror focus. Daily seizure stages and ADDs were recorded, and the kindling acquisition of LFS-S3 group and sham group were compared.

### LFS treatment for PTS at the ipsilateral hippocampus

We evaluated the effect of LFS targeted the right amygdala (primary focus) on PTS at the ipsilateral hippocampus (HP, secondary focus). Rats were randomly divided into three groups (Supplementary Fig. 2A): the Primary-HP group received daily kindling stimulation in the ipsilateral hippocampus and the Secondary-HP group received daily kindling stimulation in the ipsilateral hippocampus with the re-determined ADT after the primary focus was fully kindled; and the LFS group received LFS until the primary focus was fully kindled, then the re-determined ADT of the ipsilateral hippocampus was used for daily kindling stimulation in the ipsilateral hippocampus. The ADT of the hippocampus was measured by using similar methods as in amygdala, and 120% of the minimal intensity was used for daily stimulation thereafter. The hippocampal ADT intensity ranged from 20 to 200 μA. Daily seizure stages and ADDs were recorded, and the kindling acquisition of each group was compared.

### Immunohistochemistry

Twenty-four hours after the last kindling stimulation, rats were deeply anesthetized with pentobarbital (100 mg/kg, i.p.) and perfused transcardially with 0.9% saline followed by fixation in 4% paraformaldehyde. Rat brains and human brain tissue samples (Supplementary Table 5) were isolated and fixed in 4% paraformaldehyde at 4 °C for 24 hours and then equilibrated in 30% (w/v) sucrose. The samples were coronal sectioned (Leica, Japan) and stained for immunofluorescence for NeuN (1:500, Millipore, MAB377), and KCC2 (1:500; Abcam, ab49917) overnight at 4 °C, then rinsed with PBS and incubated with Alexa-594 or Alexa-488 conjugated secondary fluorescent antibody (1:400; Jackson ImmunoResearch) for 2 hours at room temperature. After rinsed, the sections were mounted on slides using Vectashield Mounting Media (Vector Labs) and assessed the immunofluorescence with a laser confocal microscope (LSM 510, Zeiss). The fluorescence intensity analysis and cell counting were performed by Image J software (NIH, MD, USA).

### Immunoblotting

Both sides of amygdala and hippocampi from rat brains and excised brain tissues from human (Supplementary Table 5) were homogenized in RIPA buffer (pH 7.5, in mmol/L; 20 Tris-HCl, 150 NaCl, 1 EDTA, 1% Triton-X100, 0.5% sodium deoxycholate, 1 PMSF, and 10 µg/ml leupeptin). Protein samples (50 μg/well) were separated by using SDS-polyacrylamide gel electrophoresis and transferred to a nitrocellulose membrane, which was then blocked with 5% skim milk diluted in PBS (pH 7.4) for 1 hour. Then the membranes were incubated with primary antibodies against KCC2 (1:500; Abcam) and β-tubulin (1:10000, BM1453) overnight at 4 °C. Secondary antibodies against rabbit (IRDye 800-coupled, 1:6000) or mouse (IRDye 700-coupled, 1:8000) were performed for 2 hours at room temperature, and blots were visualized with the Odyssey imaging system (LI-COR Biosciences). Digital images were quantified using densitometric measurement with Quantity-One software (Bio-Rad). The relative density was determined via comparison with the control group.

### Statistics

Data are presented as the mean ± S.E.M. Statistical comparisons were performed with SPSS (version 17.0) with appropriate methods as indicated in the figure legends. Only *p* < 0.05 was considered as a significant difference.

## Electronic supplementary material

Supplementary Information

## References

[1] Morrell F (1959). Experimental focal epilepsy in animals. Arch Neurol.

[2] Goldensohn, E. S. The relevance of secondary epileptogenesis to the treatment of epilepsy: kindling and the mirror focus. *Epilepsia* **25** Suppl 2, S156–S173 (1984). 10.1111/j.1528-1157.1984.tb05648.x 6430692

[3] Morrell F (1989). Varieties of human secondary epileptogenesis. J Clin Neurophysiol.

[4] Bortolato M (2010). Involvement of GABA in mirror focus: a case report. Epilepsy Res.

[5] Kim J (2014). Mirror focus in a patient with intractable occipital lobe epilepsy. Journal of epilepsy research.

[6] Morrell F (1985). Secondary epileptogenesis in man. Arch Neurol.

[7] Gilmore R, Morris H, Van Ness PC, Gilmore-Pollak W, Estes M (1994). Mirror focus: function of seizure frequency and influence on outcome after surgery. Epilepsia.

[8] Sampaio L, Yacubian EM, Manreza ML (2004). The role of mirror focus in the surgical outcome of patients with indolent temporal lobe tumors. Arquivos de neuro-psiquiatria.

[9] Liu Z, Gatt A, Werner SJ, Mikati MA, Holmes GL (1994). Long-term behavioral deficits following pilocarpine seizures in immature rats. Epilepsy Res.

[10] Lee SA (2015). The effect of recurrent seizures on cognitive, behavioral, and quality-of-life outcomes after 12 months of monotherapy in adults with newly diagnosed or previously untreated partial epilepsy. Epilepsy Behav.

[11] Kern R (2005). Cognitive impairment, aphasia, and seizures in a 51-year-old man. Lancet Neurol.

[12] Price MG (2009). A triplet repeat expansion genetic mouse model of infantile spasms syndrome, Arx(GCG)10 + 7, with interneuronopathy, spasms in infancy, persistent seizures, and adult cognitive and behavioral impairment. J Neurosci.

[13] Iyer MB, Schleper N, Wassermann EM (2003). Priming stimulation enhances the depressant effect of low-frequency repetitive transcranial magnetic stimulation. J Neurosci.

[14] Theodore, W. H. & Fisher, R. S. Brain stimulation for epilepsy. *Lancet Neurol* **3**, 111–118, doi:S1474442203006641 (2004). 10.1016/s1474-4422(03)00664-1 14747003

[15] Zhong K (2012). Wide therapeutic time-window of low-frequency stimulation at the subiculum for temporal lobe epilepsy treatment in rats. Neurobiol Dis.

[16] Cheng H (2015). Low-frequency stimulation of the external globus palladium produces anti-epileptogenic and anti-ictogenic actions in rats. Acta pharmacologica Sinica.

[17] Koubeissi MZ, Kahriman E, Syed TU, Miller J, Durand DM (2013). Low-frequency electrical stimulation of a fiber tract in temporal lobe epilepsy. Ann Neurol.

[18] Yang LX (2006). Unilateral low-frequency stimulation of central piriform cortex delays seizure development induced by amygdaloid kindling in rats. Neuroscience.

[19] Wang, S. *et al*. Low-frequency stimulation of cerebellar fastigial nucleus inhibits amygdaloid kindling acquisition in Sprague-Dawley rats. *Neurobiol Dis* **29**, 52–58, doi:S0969-9961(07)00184-210.1016/j.nbd.2007.07.027 (2008). 10.1016/j.nbd.2007.07.027 17904855

[20] Li H, Weiss SR, Chuang DM, Post RM, Rogawski MA (1998). Bidirectional synaptic plasticity in the rat basolateral amygdala: characterization of an activity-dependent switch sensitive to the presynaptic metabotropic glutamate receptor antagonist 2S-alpha-ethylglutamic acid. J Neurosci.

[21] Dudek SM, Bear MF (1992). Homosynaptic long-term depression in area CA1 of hippocampus and effects of N-methyl-D-aspartate receptor blockade. Proc Natl Acad Sci USA.

[22] Lesser RP (1999). Brief bursts of pulse stimulation terminate afterdischarges caused by cortical stimulation. Neurology.

[23] Khalilov I, Holmes GL, Ben-Ari Y (2003). *In vitro* formation of a secondary epileptogenic mirror focus by interhippocampal propagation of seizures. Nat Neurosci.

[24] Nardou R (2011). Neuronal chloride accumulation and excitatory GABA underlie aggravation of neonatal epileptiform activities by phenobarbital. Brain.

[25] Morrell F (1960). Secondary epileptogenic lesions. Epilepsia.

[26] Wang Y (2014). Low-frequency stimulation inhibits epileptogenesis by modulating the early network of the limbic system as evaluated in amygdala kindling model. Brain structure & function.

[27] Xu ZH (2010). Therapeutic time window of low-frequency stimulation at entorhinal cortex for amygdaloid-kindling seizures in rats. Epilepsia.

[28] Ben-Ari Y, Dudek FE (2010). Primary and secondary mechanisms of epileptogenesis in the temporal lobe: there is a before and an after. Epilepsy Curr.

[29] Cibula JE, Gilmore RL (1997). Secondary epileptogenesis in humans. J Clin Neurophysiol.

[30] Falconer MA, Kennedy WA (1961). Epilepsy due to small focal temporal lesions with bilateral independent spike-discharging foci. A study of seven cases relieved by operation. J Neurol Neurosurg Psychiatry.

[31] Rivera C (1999). The K+/Cl− co-transporter KCC2 renders GABA hyperpolarizing during neuronal maturation. Nature.

[32] Fritsch B (2010). Direct current stimulation promotes BDNF-dependent synaptic plasticity: potential implications for motor learning. Neuron.

[33] Pang PT, Teng HK, Zaitsev E, Woo NT, Sakata K, Zhen S, Teng KK, Yung WH, Hempstead BL, Lu B (2004). Cleavage of proBDNF by tPA/plasmin is essential for long-term hippocampal plasticity. Science..

[34] Wang, S., Wu, D. C., Fan, X. N. *et al*. Mediodorsalthalamic stimulation is not protective against seizures induced by amygdaloid kindling in rats. *Neurosci Lett*. **6**, 481(2), 97–101 (2010) 10.1016/j.neulet.2010.06.060 20600600

[35] Wu, D. C., Zhu-Ge, Z. B., Yu, C.Y. *et al*. Low-frequency stimulation of the tuberomammillary nucleus facilitates electrical amygdaloid-kindling acquisition in Sprague-Dawley rats. *Neurobiol Dis*. **32**(1), 151–156 (2008). 10.1016/j.nbd.2008.07.002 18675356

[36] Sun HL (2010). Mode-dependent effect of low-frequency stimulation targeting the hippocampal CA3 subfield on amygdala-kindled seizures in rats. Epilepsy Res.

[37] Paxinos, G. and Watson, C. The rat brain in stereotaxic coordinates. 6th ed., Amsterdam; Boston: Academic Press/Elsevier (2007).

[38] Racine RJ (1972). Modification of seizure activity by electrical stimulation. II. Motor seizure. Electroencephalogr Clin Neurophysiol.

[39] Engel J, Wolfson L, Brown L (1978). Anatomical correlates of electrical and behavioral events related to amygdaloid kindling. Ann Neurol.

[40] Hewapathirane DS, Burnham WM (2005). Propagation of amygdala-kindled seizures to the hippocampus in the rat: electroencephalographic features and behavioural correlates. Neuroscience research.

