# Supplementary material for: Low-frequency stimulation of the primary focus retards positive transfer of secondary focus
Source: Sci Rep. 2017 Mar 23;7:345. doi: 10.1038/s41598-017-00479-z (PMC5428430; doi:10.1038/s41598-017-00479-z)
Supplement: Supplementary file 1 — Supplementary Information [file 41598_2017_479_MOESM1_ESM.pdf]

Submitted to *Scientific Reports*

Original Article

Low-frequency stimulation of the primary focus retards positive transfer of secondary focus

Yifang Kuang[1,2#] MD, PhD, Cenglin Xu[2#] PhD, Yinxi Zhang[1#] MD, Yi Wang[2] PhD, Xiaohua Wu[1] MD, Ying Wang[2] PhD, Yao Liu[2] PhD, Kai Zhong[2] PhD, Hui Cheng[2,3] MD, PhD, Yi Guo[1] MD, PhD, Shuang Wang[1] MD, PhD, Meiping Ding [1*] MD, and Zhong Chen [1, 2, 4*] PhD

[1] Department of Neurology & Epilepsy Center, Second Affiliated Hospital, School of Medicine, Zhejiang University, Hangzhou, China; [2]Department of Pharmacology, Key Laboratory of Medical Neurobiology of the Ministry of Health of China, College of Pharmaceutical Sciences, School of Medicine, Zhejiang University, Hangzhou, China; [3]Department of Neurology, Sir Run Run Shaw Hospital, School of Medicine, Zhejiang University, Hangzhou China; [4]Collaborative Innovation Center for Diagnosis and Treatment of Infectious Diseases, First Affiliated Hospital, School of Medicine, Zhejiang University, Hangzhou, China;

[#]These authors contributed equally to this work.

*Correspondence to: Professor Zhong Chen, PhD and Professor Meiping Ding, MD

E-mail address: chenzhong@zju.edu.cn, meipingd@163.com

Tel & Fax: +86-571-88208228;

Supplementary Materials

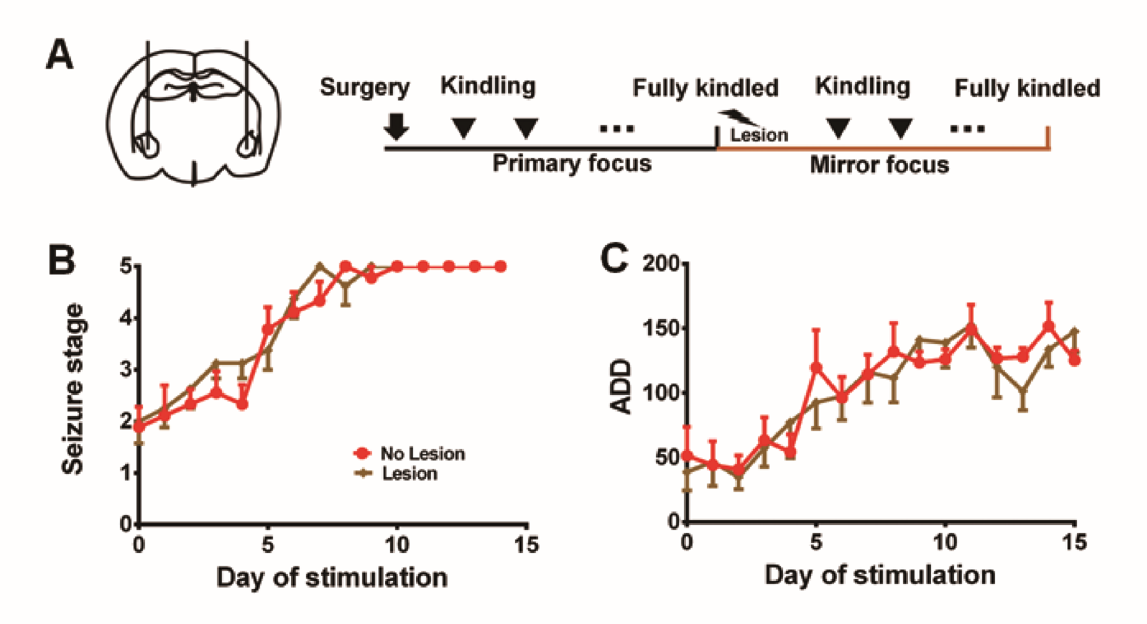

Supplementary Figure 1. The fully-kindled primary focus is not necessary for the promotion of PTS. (A) Schematic of experiment. **(B** and **C)** The development of behavioral stages **(B)**, and ADD **(C)** during kindling acquisition of the mirror focus when the primary site was fully kindled and then electrically lesioned (n = 9 for the Lesion group, n = 8 for the Mirror-focus group).

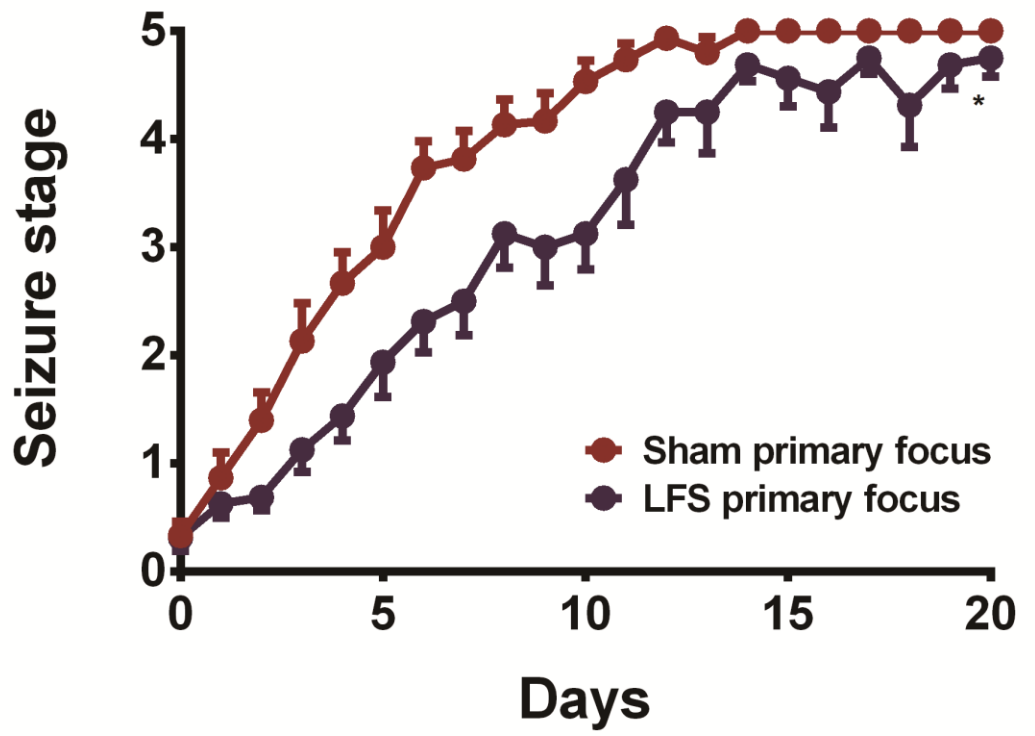

Supplementary Figure 2. LFS retarded the kindling acquisition of the primary focus. LFS at the primary focus could retard the progression of behavioral seizure stage in the kindling acquisition of the primary focus. *$p < 0.05$, compared with the Sham group (n=16 for LFS group, n=15 for sham group). Two-way ANOVA followed by LSD *post hoc* tests were used for statistical analysis

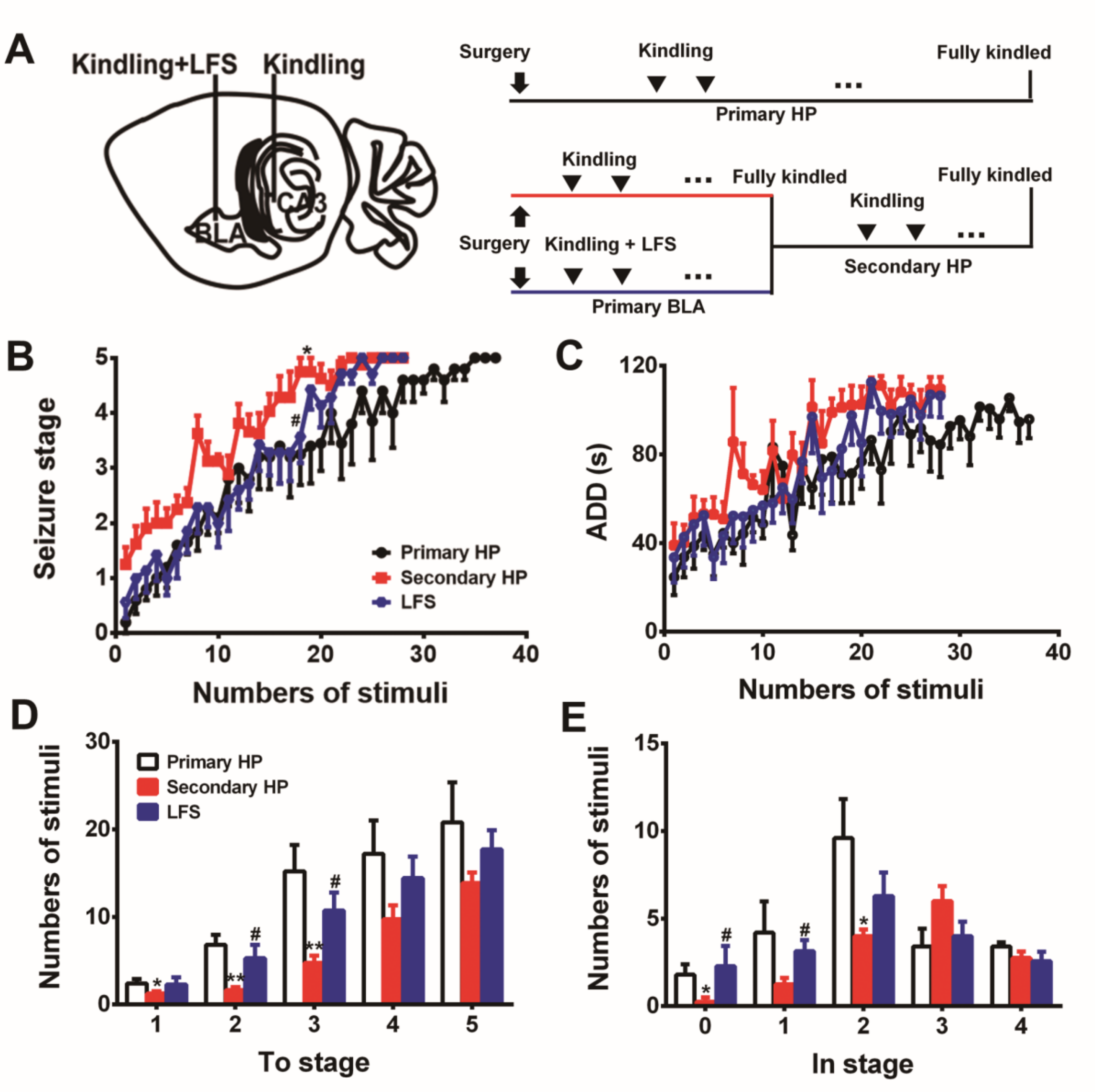

Supplementary Figure 3. LFS retarded PTS at the ipsilateral hippocampus. (A) Schematic of experiments. **(B-E)** Effect of LFS on behavioral stages **(B)**, ADD **(C)**, days of stimulation required to reach each stage **(D)**, and days of stimulation in each stage **(E)** during kindling acquisition of the ipsilateral hippocampus when the primary focus was fully kindled (n = 5 for the Primary-HP group, n = 8 for the Secondary-HP group, and n = 7 for the LFS group). *$p < 0.05$, **$p < 0.01$ compared with the Primary HP group; #p <

0.05 compared with the Secondary-HP group. Two-way ANOVA followed by LSD *post hoc* tests were used for statistical analysis of **B** and **C**. One-way ANOVA followed by LSD *post hoc* tests were used for statistical analysis of **D** and **E**.

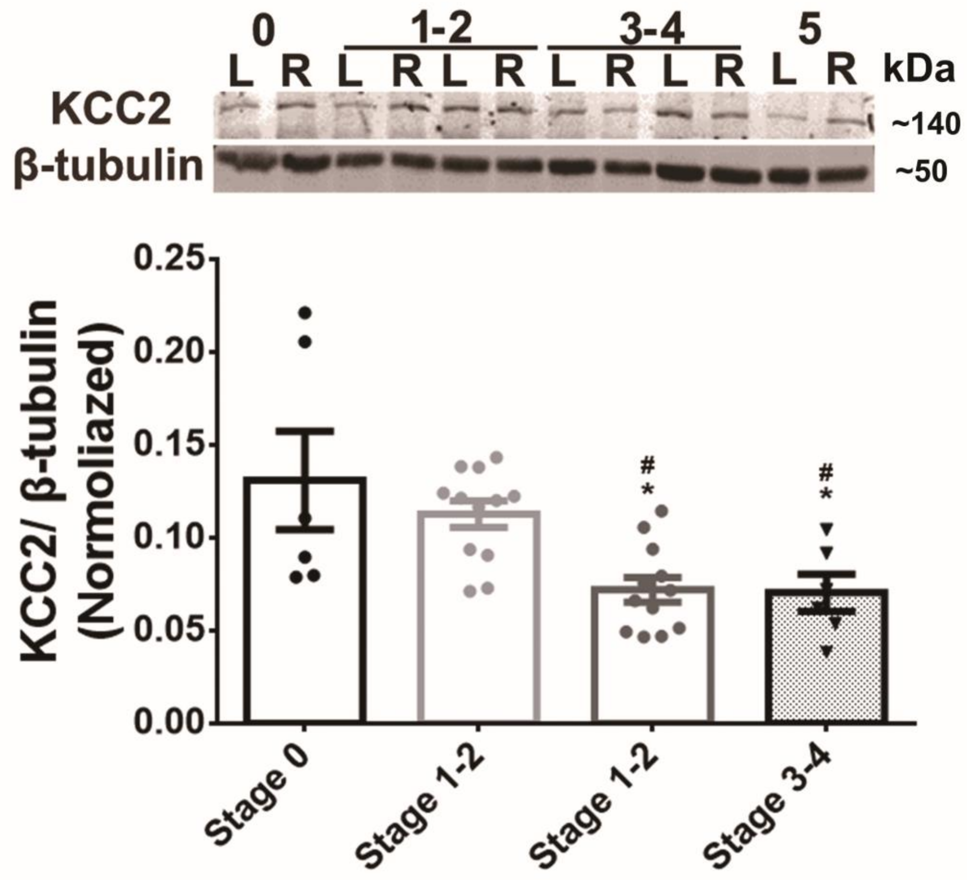

Supplementary Figure 4. Expression of KCC2 in different stages of primary kindling acquisition. Representative western-blot image of the expression of KCC2 and densitometric analysis at different seizure stages of the primary kindling acquisition. *$p < 0.05$: compared with the stage 0; #$p < 0.05$: compared with the stage 1-2. One-way ANOVA followed by LSD *post hoc* tests were used for statistical analysis.

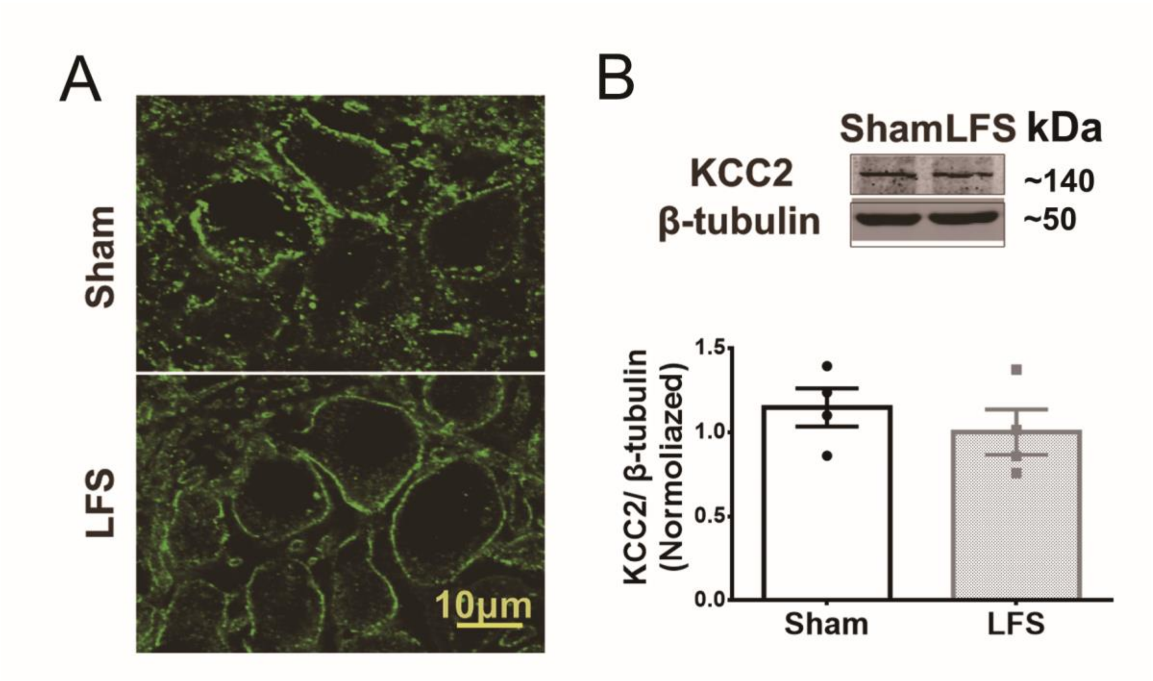

Supplementary Figure 5. LFS does not change the expression of KCC2 in normal rats. (A) Expression of KCC2 immunoreactivity was not significantly different near the cell membrane between tissue of the Sham group and the LFS group. **(B)** Representative western-blot image of the expression of KCC2 and densitometric analysis indicating no significant change between the sham and the LFS groups. Data were tested by Mann-Whitney U tests.

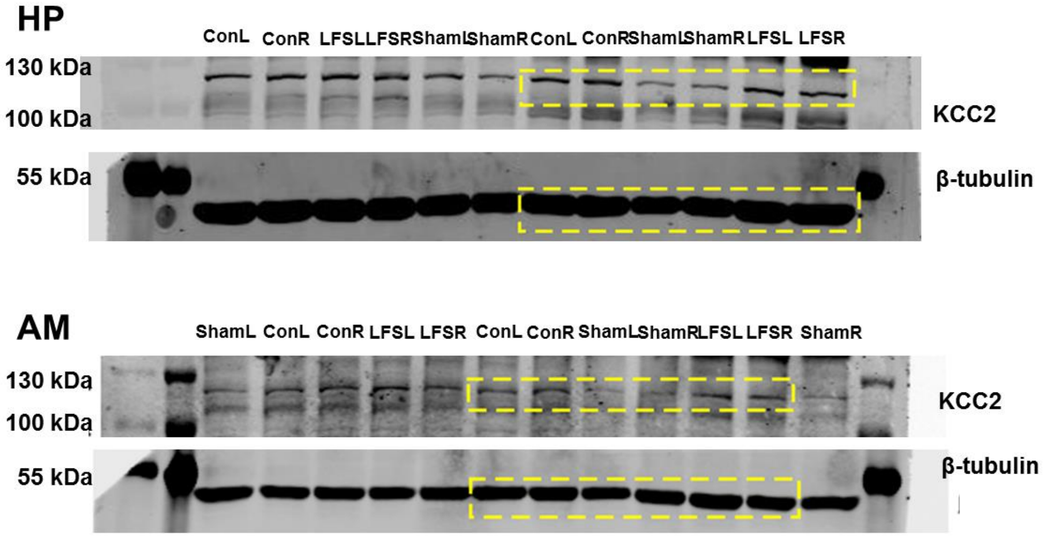

Supplementary Figure 6. Full length western blot of Figure 5C.

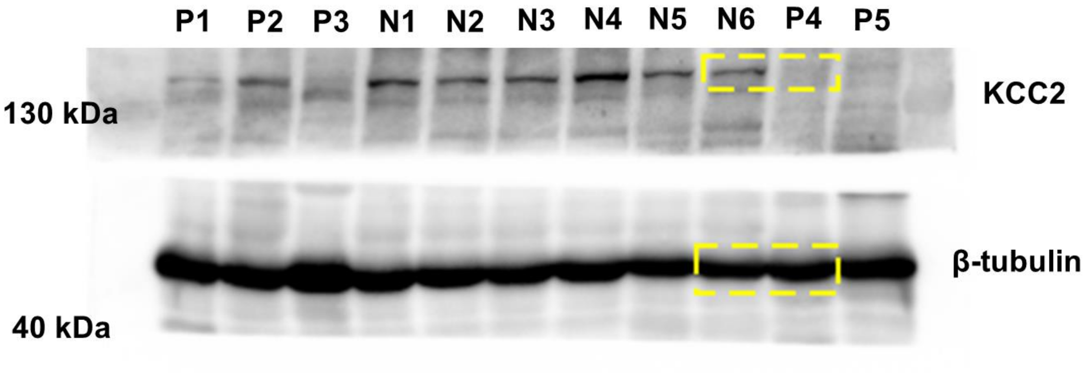

Supplementary Figure 7. Full length western blot of Figure 6B.

Supplementary table 1. Clinical characteristics of PT patients

PT group

Subjects	Gender (M/F)	Age (year)	Course (year)	Resection Tissue	Seizure Type	Seizure Frequency (ever month)	Pathological Diagnosis	Seizure-free after Surgery	AEDs after the Surgery
P1	M	38	＞20	TNr(Amygdala and Hippocampus)	Complex focal to generalized seizures	6~7	FCD	N	LTG, CBZ
P2	M	22	＞7	TNr(Amygdala and Hippocampus)	Complex focal to generalized seizures	0~3	FCD, G	N	OXC, LEV
P3	M	23	0.5	Left temporal lobe	Complex focal to generalized seizures	Unknown	GG	Y	VPA
P4	F	14	＞1	TNr(Posterior middle temporal gyrus)	Focal seizures(Motor)	1~2	DNT	Y	OXC
P5	F	27	27	TNr(Amygdala and Hippocampus)	Complex focal to generalized seizures	2~3	G	Y	LTG, LEV
P6	F	26	12	TNr(Amygdala and Hippocampus)	Complex focal to generalized seizures	2~5	HS, G	Y	CBZ, LTG

Abbreviations: P, Positive-transfer group patient; F, female; M, male; AEDs, antiepileptic drugs; VPA, valproate; CBZ, carbamazepine; LTG, lamotrigine; LEV, levetiracetam; OXC, oxcarbazepine; CZP, clonazepam; l, left; r, right; TN, temporal neocortex; G, gliosis; DNT, dysembryoplastic; FCD, focal cortical dysplasia; GG, ganglioglioma; HS, hippocampal sclerosis; D: days; Y: yes; N: no.

Supplementary table 2. Clinical characteristics of Non-PT patients

Non-PT group

Subjects	Gender (M/F)	Age (year)	Course (year)	Resection Tissue	Seizure Type	Seizure Frequency(every month)	Pathological Diagnosis	Seizure-free after Surgery	AEDs after the Surgery
N1	M	43	37	TNr(Amygdala and Hippocampus)	Complex focal to generalized seizures	Unknown	FCD, G	Y	CZP
N2	M	25	3	TNr(Posterior lateral fissure)	Complex focal to generalized seizures	2~4	GG	Y	OXZ, LTG
N3	F	26	12	TNr(Amygdala and Hippocampus)	Complex focal seizures	8~12	G	Y	OXC, LEV
N4	F	39	＞30	TNr(Amygdala and Hippocampus)	Complex focal seizures	1~2	FCD	Lost to follow up	
N5	F	40	＞3	TNr(Amygdala and Hippocampus)	Complex focal seizures	3~4	HS, G	Y	Drug withdraw
N6	F	25	>9	TNr(Middle temporal gyrus, 5.5cm from temporal pole, 3cm of Hippocampus and lateral amygdala)	Complex focal seizures	1	G	Y	OXZ, LEV
N7	F	27	>3	Anterior right temporal lobe	Complex focal seizres	>4	HS	Y	LTG

Abbreviations: N, Non-positive-transfer group patient; F, female; M, male; AEDs, antiepileptic drugs; VPA, valproate; CBZ, carbamazepine; LTG, lamotrigine; LEV, levetiracetam; OXC, oxcarbazepine; CZP, clonazepam; l, left; r, right; TN, temporal neocortex; G, gliosis; DNT, dysembryoplastic; FCD, focal cortical dysplasia; GG, ganglioglioma; HS, hippocampal sclerosis; D: days; Y: yes; N: no.

Supplementary table 3. Average stats for PT and Non-PT groups.

Sex	Groups	Age (year)	Course (year)	Seizure frequency (per month)	Percentage of sclerosis
Male	PT	27.67 ±5.18	9.167 ±5.73	5.00 ±2.00	0
	Non-PT	34.00 ±9.00	20.00 ±17.00	4.00*	0
	Significance	No	No	No	/
Female	PT	22.33 ±4.18	13.33 ±7.54	3.33 ±0.88	33%
	Non-PT	31.40 ±3.33	11.40 ±4.97	4.60 ±1.93	40%
	Significance	No	No	No	/

*Seizure frequency was recorded in only one patient.

Supplementary table 4. The degree of hippocampus sclerosis in PT and Non-PT patients.

	Pathology	MRI	Grade
P6	moderate neuronal loss and gliosis	T2:hyperintense signal in TNr	1
N5	moderate neuronal loss and gliosis	T2:hyperintense signal in TNr	1
N7	moderate neuronal loss and gliosis	T2:hyperintense signal in TNr	1

The scoring system refers to neuronal cell loss and gliosis:0: no obvious neuronal loss or moderate astrogliosis only; 1: moderate neuronal loss and gliosis; 2: sever neuronal loss (majority of neurons lost) and fibrillary astrogliosis.

Supplementary table 5. Patients sample size for immunohistochemistry and western blot

Experiments	Gender	PT	Non-PT
	Male	P1 P2	N1
		P3	N2
Western Blot	Female	P4	N3 N4 N5
		P5	N6
	Male	P1	N2
		P2 P3	
Immunohistochemistry			
	Female	P5	N3
		P6	N7
